# Magnolin Inhibits Paclitaxel-Induced Cold Allodynia and ERK1/2 Activation in Mice

**DOI:** 10.3390/plants12122283

**Published:** 2023-06-12

**Authors:** Nari Kim, Geehoon Chung, So-Ri Son, Jae Hyun Park, Young Hyun Lee, Keon-Tae Park, Ik-Hyun Cho, Dae Sik Jang, Sun Kwang Kim

**Affiliations:** 1 Department of Science in Korean Medicine, Graduate School, Kyung Hee University, Seoul 02447, Republic of Korea; ktysong702@naver.com (N.K.); dockr79@naver.com (Y.H.L.); ihcho@khu.ac.kr (I.-H.C.); 2 Department of Physiology, College of Korean Medicine, Kyung Hee University, Seoul 02447, Republic of Korea; geehoon.chung@khu.ac.kr; 3 Department of Biomedical and Pharmaceutical Sciences, Graduate School, Kyung Hee University, Seoul 02447, Republic of Korea; allosori@khu.ac.kr (S.-R.S.); pgt940116@khu.ac.kr (K.-T.P.); 4 Department of East-West Medicine, Graduate School, Kyung Hee University, Seoul 02447, Republic of Korea; blue-0523@daum.net; 5 Department of Convergence Medical Science, College of Korean Medicine, Kyung Hee University, Seoul 02447, Republic of Korea

**Keywords:** paclitaxel, neuropathic pain, magnolin, allodynia, ERK

## Abstract

Chemotherapy-induced peripheral neuropathy (CIPN) is a common side effect of anti-cancer drugs. The main symptoms often include sensory disturbances and neuropathic pain, and currently there is no effective treatment for this condition. This study aimed to investigate the suppressive effects of magnolin, an extracellular signal-regulated kinase (ERK) inhibitor substance derived from a 95% EtOH extract of the seeds of *Magnolia denudata*, on the symptoms of CIPN. A taxol-based anti-cancer drug paclitaxel (PTX) was repeatedly injected (2 mg/kg/day, total 8 mg/kg) into mice to induce CIPN. A neuropathic pain symptom was assessed using a cold allodynia test that scores behaviors of licking and shaking paw after plantar administration of acetone drop. Magnolin was administered intraperitoneally (0.1, 1, or 10 mg/kg) and behavioral changes to acetone drop were measured. The effect of magnolin administration on ERK expression in the dorsal root ganglion (DRG) was investigated using western blot analysis. The results showed that the repeated injections of PTX induced cold allodynia in mice. Magnolin administration exerted an analgesic effect on the PTX-induced cold allodynia and inhibited the ERK phosphorylation in the DRG. These results suggest that magnolin could be developed as an alternative treatment to suppress paclitaxel-induced neuropathic pain symptoms.

## 1. Introduction

Paclitaxel (PTX) is a chemotherapy agent derived from *Taxus brevifolia* and is widely used for the treatment of various cancer diseases, including breast, lung, ovarian, and esophageal tumors [1]. Many patients treated with PTX show side effects of peripheral neuropathy manifested by sensory disturbances and pain in the extremities. According to reports from clinics, more than 80% of patients experienced a tingling sensation and more than 60% of patients had a cold sensation [2]. As in the case of neuropathic pain caused by other diseases or lesions of the sensory nervous system [3], symptoms of chemotherapy-induced peripheral neuropathy (CIPN) are not relieved by conventional analgesics. The management of neuropathic pain symptoms became an important problem for the maintenance of chemotherapy [4], as cancer patients often abandon chemotherapy because of this side effect. There remains an unmet need for the treatment of CIPN symptoms in clinics.

An important molecule responsible for the neuropathic pain condition is mitogen-activated protein kinase (MAPK), such as extracellular signal-regulated kinase (ERK). ERK1 and ERK2 (ERK1/2) belong to an evolutionarily conserved group of serine/threonine protein kinases, and they play important roles in the change of the nervous system in various diseases. Previous studies have reported that phosphorylation of ERK (pERK) is increased in the sensory nervous system in neuropathic pain conditions, resulting in the manifestation of allodynia and hyperalgesia symptoms [5,6,7,8,9]. It has been also reported that inhibiting the ERK is effective in relieving pain in animal models of this neuropathic pain, suggesting the ERK could be an important therapeutic target for the treatment of neuropathic pain [10].

The flower buds of *Magnolia denudata* Desrousseaux (Magnoliae Flos, Magnoliaceae) have traditionally been used as a herbal medicine to treat various immunological conditions and pain symptoms [11]. Magnolin, the major tetrahydrofurofuranoid lignan from *M*. *denudata,* has been isolated from “Shin-i”, a collective term used in Korea to refer to the flower buds of *M*. *denudata*, *M. biondii*, *M*. *kobus*, and *M*. *sprengeri* [12]. Magnolin could relieve pain symptoms in the various pain models, including chronic constriction injury (CCI), complete Freund’s adjuvant (CFA), oxaliplatin, and chronic inflammatory visceral pain (CIVP) [8,13,14,15] by inhibition of ERK activation. However, whether magnolin could attenuate the neuropathic pain and the changes in ERK activation induced by paclitaxel administration have not been studied.

Previous animal studies have confirmed that PTX administration induces neuropathic pain-like symptoms including cold allodynia in mice [16], and an increase in total expression and phosphorylation of ERK in the dorsal root ganglion (DRG) is critically involved in this neuropathic pain condition [17]. In this study, we investigated the analgesic efficacy of magnolin on the cold allodynia symptom induced by PTX in mice. Further, we analyzed the PTX-induced increase in ERK phosphorylation in the DRG and its inhibition by the magnolin treatment.

## 2. Results

### 2.1. Isolation of Magnolin

**Figure 1:**
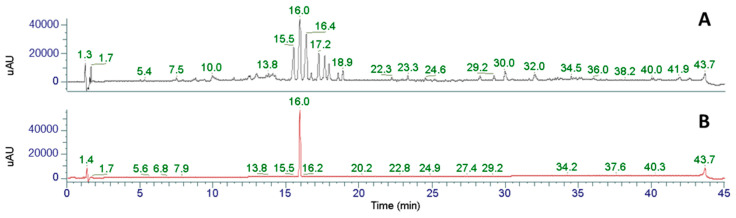
UHPLC-PDA chromatogram of the 70% EtOH extract from *M*. *denudata* (**A**) and magnolin ((**B**); *R_t_* 16.0 min). Kobusin and aschantin were detected at *R*_t_ 15.5 and 16.4, respectively. Data collected by Thermo Vanquish UHPLC system equipped with Thermo Hypersil GOLD column (1.9 μm, 150 mm× 2.1 mm I.D.). The mobile phase was subjected to a series of linear gradients with a flow rate of 0.3 mL/min: 0–3 min, 10% B; 3–35 min, 100% B; 35–42 min, 100% B; 42–43 min 10% B.

**Figure 2:**
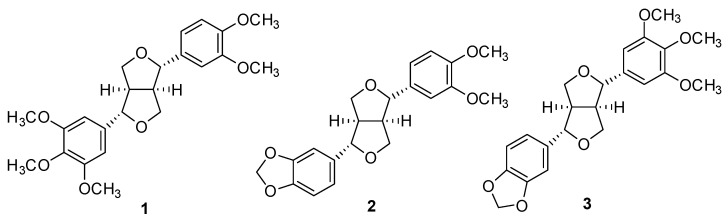
Chemical structures of magnolin (**1**), kobusin (**2**), and aschantin (**3**) isolated from the seeds of *M. denudata.*

We initially screened an 95% EtOH extract from the seeds of *M*. *denudata* using LC-PDA-MS and identified three tetrahydrofurofuranoid lignans, magnolin (*R*_t_ 16.0 min), kobusin (*R*_t_ 15.5 min), and aschantin (*R*_t_ 16.4 min) as major constituents (Figure 1 and Figure 2).

Thus, repetitive chromatography was employed to isolate the most abundant magnolin on a gram scale from the seeds of *M. denudata* in order to investigate the analgesic efficacy of it on the cold allodynia symptom induced by PTX in mice. The chemical structure of (+)-magnolin (Figure 2) was identified using specific rotation, ^1^H-NMR, ^13^C-NMR, and LC-MS measurements and compared to published values (Figures S1–S3) [18]. The purity of (+)-magnolin obtained in this study was determined to be >98% by NMR and LC-PDA-MS experiments.

### 2.2. Cold Allodynia in Mice after Injection of PTX

**Figure 3:**
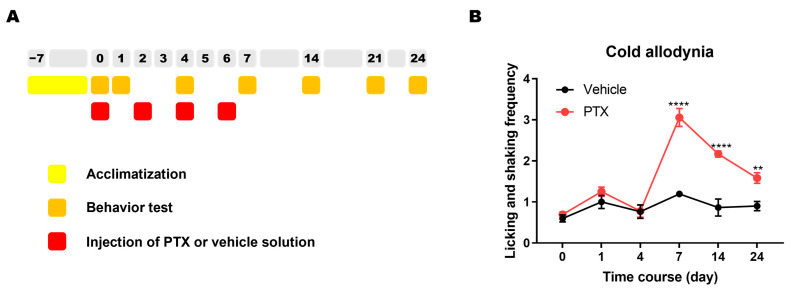
Induction of cold allodynia symptom following repetitive injection of PTX. (**A**) Experimental schedule. (**B**) Increased behavioral response to plantar application of acetone drop in mice treated with PTX. (Vehicle group, *n* = 5; PTX group, *n* = 6; **** *p* < 0.0001, day 7 and 14; ** *p* < 0.01, day 24; Two-way repeated measures ANOVA followed by Sidak’s multiple comparisons test, compared to the control group).

Animals were randomly assigned to the PTX or the control group. PTX (2 mg/kg) was intraperitoneally administered to PTX group mice every other day (days 0, 2, 4, and 6) to induce symptoms of CIPN (Figure 3A). Control group mice were injected with vehicle solution with the same schedule. An averaged frequency of the behavioral responses of licking or shaking the hind paw in response to the administration of acetone drop was measured to assess cold allodynia, a representative symptom of the PTX-induced CIPN. The acetone drop tests were repeated before (day 0) and after the PTX injection (days 1, 7, 14, and 24). As reported in previous studies [19,20,21], the behavioral response against the administration of acetone drop to the plantar surface was increased in mice treated with PTX. The PTX-treated mice showed a significantly higher frequency of licking and shanking behavior compared to vehicle-treated mice from day 7 to day 24 (Figure 3B).

### 2.3. Analgesic Effect of Magnolin on PTX-Induced Cold Allodynia

**Figure 4:**
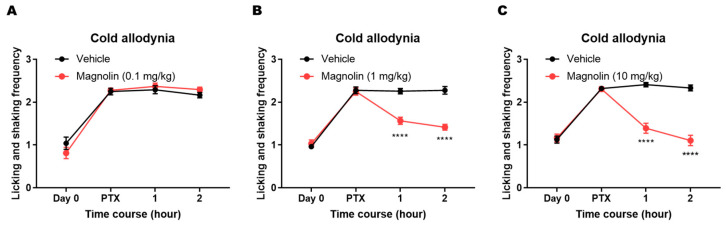
Attenuation of PTX-induced cold allodynia by magnolin treatment. (**A**) No significant behavioral change in PTX group mice treated with 0.1 mg/kg magnolin (Vehicle group, *n* = 8; Magnolin group, *n* = 9). (**B**) Significant attenuation of PTX-induced cold allodynia in mice treated with 1 mg/kg magnolin (Vehicle group, *n* = 9; Magnolin group, *n* = 10). (**C**) Significant attenuation of PTX-induced cold allodynia in mice treated with 10 mg/kg magnolin (Vehicle group, *n* = 13; Magnolin group, *n* = 14). Behavior levels at baseline (before PTX, e.g., day 0) were displayed with each test result. Statistical comparisons were performed at each time point (**** *p* < 0.0001; Two-way repeated measures ANOVA followed by Sidak’s multiple comparisons test, compared to the vehicle-treated control group).

To assess the analgesic effect of magnolin on cold allodynia, PTX group mice were randomly divided into experimental or control groups and intraperitoneally injected with three different doses of magnolin (0.1, 1, or 10 mg/kg) or vehicle solution. Based on the prior behavioral evaluation, the tests were performed on a day between 7 to 14 when the cold allodynia was the most severe. Behavioral tests were performed before and after the magnolin treatment (1 and 2 h). The lowest dose of 0.1 mg/kg did not affect cold allodynia (Figure 4A). Significant analgesic effects appeared in the groups treated with doses of 1 mg/kg (Figure 4B) or 10 mg/kg (Figure 4C). The analgesic effect of magnolin treatment on PTX-induced cold allodynia was maintained for at least 2 h (Figure 4B,C).

### 2.4. Effect of Magnolin Treatment on ERK Phosphorylation in the DRG of PTX-Treated Mice

**Figure 5:**
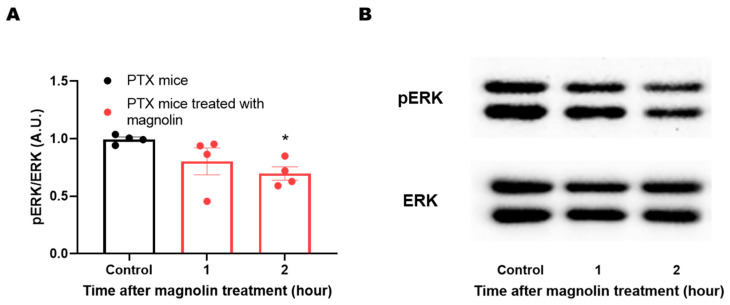
Attenuation of PTX-induced ERK activity by magnolin treatment. (**A**) Significantly lower levels of pERK/ERK ratio (arbitrary unit, %) in the magnolin-treated mice compared to the untreated group (*n* = 4 per group; each data point pooling 3 subject samples; animal subject *N* = 12 per group) (* *p* < 0.05; Kruskal–Wallis test followed by Dunn’s multiple comparisons test, compared to the control group). (**B**) Protein expression demonstrated by western blot data.

Phosphorylation of ERK in the sensory nervous system is critically involved in the manifestation of various neuropathic symptoms, including cold allodynia. Previous studies have shown that magnolin directly targets ERKs and inhibits their signaling. To confirm whether the magnolin treatment could attenuate pERK level in the mouse DRG, samples were acquired from the PTX group on day 14 when the distinct cold allodynia symptom appeared in PTX-treated mice. The DRG samples were collected from separate groups based on the time elapsed after the magnolin treatment (1 and 2 h). Magnolin treatment attenuated the ERK activity in the DRG, shown by the significantly lower levels of pERK/ERK ratio in the groups treated with magnolin compared to the untreated PTX group (Figure 5A,B).

## 3. Discussion

Among the various side effects of anticancer drugs, symptoms of CIPN are known to be a major reason for patients to abandon chemotherapy. CIPN patients often suffer from chronic sensory disturbances, including pain symptoms. Since these symptoms do not respond well to conventional analgesic drugs, there is an unmet need in the clinics for the management of the symptoms. In attempts to develop novel therapeutics, one strategy is to investigate the analgesic properties of plant-derived substances. Many medicinal plants that have been used in traditional medicine include active ingredients that could exert analgesic effects. For example, narcotic analgesics are derived from opium and have been used for thousands of years as a potent pain treatment. The strategy of isolating analgesic candidates from plants with known empirical efficacy can help in the development of successful analgesics for CIPN [22]. However, a single medicinal substance typically contains numerous active ingredients, and which of these are selected and tested as candidates is the key to successful candidate identification.

In this study, we focused on the change of the ERK1/2 activation in PTX-induced neuropathic pain state. In the nervous system, changes in ERK1/2 phosphorylation are critically involved in the development and maintenance of chronic neuropathic pain. Upon nerve injury, ERK1/2 is activated in neurons and glia in the spinal cord and DRG [23] as well as in brain regions [24]. Activated ERK1/2 in turn affects long-term potentiation of neuronal activity in the nervous system, contributing to central sensitization [23,24,25,26]. PTX is known to increase the activity of ERK1/2 in the DRG [27], similar to neuropathic pain with other causes [17].

Recent investigations have revealed the bioactive compounds from plant sources as selective ERK1/2 activation inhibitors [28]. This has led to the discovery of some naturally occurring phenolic compounds with ERKs-inhibitory properties. Notably, catechol, usually found in fruits and vegetables, inhibits the ERK2/c-Myc signaling axis, resulting in a decrease in lung cancer tumors [29]. Similarly, vitisin A, a resveratrol tetramer from *Vitis vinifera* roots, inhibits LPS-induced ERK1/2 in RAW 264.7 cells [30]. Rocaglamides, derived from the genus *Aglaia*, inhibit proliferation by downregulating ERK activity [31]. In addition, naturally occurring lignans have been shown to inhibit the activation of the ERK1/2 pathway. For instance, 7-Hydroxymatairesinol and 7-hydroxymatairesinol 2 from *Picea abies* have anti-inflammatory effects by reducing ERK phosphorylation [32]. Schisandrin C, a lignan found in *Schisandra chinensis*, also exhibits anti-inflammatory activity by blocking phosphorylated ERK1/2 [33].

Magnolia species, including *M*. *fargesii*, *M*. *denudata*, *M*. *biondii*, and *M*. *sprengeri* have been identified as a rich source of lignans. In particular, *M. denudata*, a medicinal plant used in traditional medicine to treat pain diseases, inflammation, and allergic symptoms [34,35], contains several major lignans, including magnolin, aschantin, kobusin, fargesin, and 3,4,3′4′-tetramethoxy-9,7′-dihydroxy-8,8′,7.O.9′-lignans [18]. Among several candidates, we selected magnolin, a substance that has been studied in animal models of various pain diseases [8,13,14,15]. With its inhibitory action on ERK activation [36], magnolin could modulate the production of various pain-related signaling molecules, such as tumor necrosis factor-α [37] and nitric oxide [38]. In addition, the anticancer effects of magnolin have been tested as well in animal models of lung, colorectal, breast cancer, and melanoma [39,40,41,42,43]. Considering that the ideal treatment for CIPN should not aggravate underlying cancer, this mechanism of action is advantageous for the development of new therapeutics. Given that the ERK activation is a common mechanism in both cancer tumorigenesis and CIPN [44,45], it will be interesting to investigate whether magnolin not only inhibits CIPN but also synergizes with the anticancer effects of individual chemotherapeutic agents.

As reported in previous studies, various types of sensory neuropathy symptoms have different mechanisms so the efficacy of specific therapeutics on each symptom can vary depending on the mechanistic action of the substance used [46,47,48,49]. In this study, cold allodynia was used as a read-out of the successful induction of painful peripheral neuropathy and the analgesic efficacy of the magnolin treatment. Patients of CIPN experience an innocuous cooling sensation as severe pain, and this symptom can be observed with various animal models of CIPN. A shift in the activity of sensory neurons contributes to this cold allodynia, including changes in DRG neurons responsible for ascending sensory transmission [50,51,52,53]. A previous study has reported that ERK phosphorylation was increased in an animal model of CIPN, and inhibition of ERK ameliorated neuropathic pain [53]. Although the study was performed using different rodent species (rat) and the chemotherapeutic agent (oxaliplatin), the results are consistent with our study. We found that magnolin treatment could inhibit the ERK phosphorylation in the DRG of mice treated with PTX, which attenuated the CIPN symptom, e.g., cold allodynia. 

The ERK activation involves various upstream and downstream signaling pathways and plays a pivotal role in the change of cellular functions [54]. Although this study did not explore the detailed downstream of ERK change in the CIPN, previous studies have reported the involvement of signaling pathways of ERK activation in the development and maintenance of pain symptoms [45,55]. It is unclear whether the action of magnolin to inhibit ERK1/2 also affects the cold tolerance of *M. denudata*. *M. denudata* can survive in low or freezing temperature conditions and is known to reduce the adverse effects of cold stress through multiple adaptive mechanisms [56]. A recent study investigated the physiological mechanisms involved in the long-term cold acclimation of *M. denudata* and revealed the involvement of various transcription factors [57]. The study broadly discussed changes in the expression of genes involved in plant hormones, carbohydrate metabolism, cold-related transcription factors, and antioxidation mechanisms. Given that phosphorylation of ERK1/2 is involved in a variety of intracellular signaling, it is likely that magnolin, which is of interest to us, may also be involved in the cold resistance of *M. denudata* through ERK1/2 and its downstream targets. Although we only investigated ERK1/2 inhibition in the DRG of experimental animals, magnolin could be closely involved in many of the other cold tolerance mechanisms mentioned above. Linking the mechanisms of the relief from cold allodynia in animals with mechanisms of cold tolerance in plants may provide new clues in future studies.

## 4. Materials and Methods

### 4.1. Plant Material

The seeds of *M. denudata* Desr. (Magnoliaceae) were collected at Irwon-dong, Gangnam-gu, Seoul, Republic of Korea, in March 2019. The origin of the plant was authenticated by Prof. Dae Sik Jang, one of the authors. A voucher of the specimen (MADE-2019) has been deposited in the Natural Product Medicine Laboratory, College of Pharmacy, Kyung Hee University.

### 4.2. UHPLC-PDA-MS Analysis

To identify the main components of *M*. *denudata*, a UHPLC-PDA-MS analysis was performed. The Thermo Vanquish UHPLC system was used along with the LTQ-XL-MS^n^ and Thermo Hypersil GOLD column (1.9 μm, 150 mm × 2.1 mm I.D.). The mobile phase was subjected to a series of linear gradients with a flow rate of 0.3 mL/min: 10% B from 0 to 3 min, 100% B from 3 to 35 min, 100% B from 35 to 42 min, and 10% B from 42 to 43 min. The extract was dissolved in MeOH and filtered using a PTFE filter (0.2 μm) at a concentration of 25 mg/mL. Additionally, standard solutions of the isolated compounds were also analyzed to verify the retention time. Each standard solution was dissolved in methanol containing 10% DMSO at a concentration of 0.5 mg/mL and filtered.

### 4.3. Isolation of Major Lignans from M. denudata

The seeds of *M. denudata* (1.14 kg) were refluxed twice in 95% ethanol (18 L) for 2 h. The solution was subsequently filtered through a Whatman No. 2 paper filter and concentrated by rotary evaporation at 45 °C under decreased pressure. The extract (210 g) was partitioned with H_2_O and ethyl acetate (EA) to produce H_2_O- and EA-soluble fractions. The EA-soluble fraction (36.68 g) was submitted to Diaion-HP20 column chromatography (CC) with a gradient solvent system (acetone/H_2_O = 4/6 ~ 10/0), yielding 22 fractions (F1–F22). Fraction F8 (11.15 g) was then chromatographed over Sephadex LH-20 with methylene chloride (MC) and separated into seven fractions (F8-1–F8-7). Finally, fraction F8-2 was separated by silica gel CC using a stepwise gradient mixture (MC/MeOH = 10/0 ~ 0/10), leading to the purification of (+)-magnolin (6.8 g; 0.6%) and kobusin (64.4 mg). Fraction F8-3 was chromatographed over silica gel using a gradient solvent system (MC/MeOH = 10/0 ~ 0/10) to obtain aschantin (77.4 mg).

#### 4.3.1. (+)-Magnolin (1)

White powder: ${\left[\alpha \right]}_{D}^{20}$ + 63.7 (c 0.01, CHCl_3_); ^1^H-NMR (500 MHz, CDCl_3_) *δ*_H_ 6.89 (1H, d, *J* = 2.0 Hz, H-2′), 6.86 (1H, dd, *J* = 8.0, 2.0 Hz, H-6′), 6.83 (1H, d, *J* = 8.0 Hz, H-5′), 6.56 (2H, s, H-2″ and 6″), 4.75–4.71 (2H, m, H-2 and 6), 4.31–4.23 (2H, m, H-4eq and 8eq), 3.93–3.83 (2H, m, H-4ax and 8ax), 3.88 (3H, s, -OCH_3_), 3.86 (9H, s, -OCH_3_), 3.82 (3H, s, -OCH_3_), 3.11–3.06 (2H, m, H-1 and 5); ^13^C NMR (125 MHz, CDCl_3_) *δ*_C_ 153.6 (C-3″ and 5″), 149.4 (C-1″), 148.8 (C-3′), 137.6 (C-4″), 136.9 (C-1″), 133.6 (C-1′), 118.4 (C-6′), 111.2 (C-5′), 109.3 (C-2′), 102.9 (C-2″ and 6″), 86.1 (C-2), 85.9 (C-6), 72.2 (C-4), 71.9 (C-8), 61.0, 56.4, 56.1, 54.5 (-OCH_3_), 54.3; ESI-LTQ-MS (positive mode) *m*/*z* 417 [M+H]^+^ (calcd for C_23_H_29_O_7_, 417.4).

#### 4.3.2. Kobusin (2)

White powder: ^1^H-NMR (500 MHz, CDCl_3_) *δ*_H_ 6.89 (1H, d, *J* = 2.0 Hz, H-2′), 6.86 (1H, dd, *J* = 8.0, 2.0 Hz, H-6″), 6.84 (1H, d, *J* = 2.0 Hz, H-2″), 6.83 (1H, d, *J* = 8.0 Hz, H-5″), 6.80 (1H, dd, *J* = 8.0, 2.0 Hz, H-6′), 6.77 (1H, d, *J* = 8.0 Hz, H-5′), 6.55 (2H, s, H-2″ and 6″), 5.94 (2H, s, -OCH_2_O-), 4.72 (2H, t, *J* = 4.5 Hz, H-2 and 6), 4.23 (2H, ddd, *J* = 12.0, 6.5, 6.5 Hz, H-4eq and 8eq), 3.88–3.85 (2H, m, H-4ax and 8ax), 3.88 (3H, s, -OCH_3_), 3.86 (3H, s, -OCH_3_), 3.10–3.00 (2H, m, H-1 and 5).

#### 4.3.3. Aschantin (3)

White powder: ^1^H-NMR (500 MHz, CDCl_3_) *δ*_H_ 6.84 (1H, d, *J* = 2.0 Hz, H-2′), 6.79 (1H, dd, *J* = 8.0, 2.0 Hz, H-6′), 6.77 (1H, d, *J* = 8.0 Hz, H-5′), 6.55 (2H, s, H-2″ and 6″), 5.94 (2H, s, -OCH_2_O-), 4.72 (2H, t, *J* = 4.5 Hz, H-2 and 6), 4.25 (2H, ddd, *J* = 12.0, 9.0, 6.5 Hz, H-4eq and 8eq), 3.90–3.85 (2H, m, H-4ax and 8ax), 3.85 (6H, s, -OCH_3_), 3.82 (3H, s, -OCH_3_), 3.10–3.00 (2H, m, H-1 and 5).

### 4.4. Experimental Animals

Adult C57/BL6J mice (male, 18–21 g, 6 weeks old; Deahan Biolink, Chungbuk, Korea) were provided with water and food ad libitum. All the animals were randomly separated and housed four to five per cage. The animal facility was maintained with a temperature of 23 ± 2 °C and humidity of 60–70% on a 12-h light-dark cycle (a light cycle; 08:00–20:00). The animals were adapted to the environment for a week before conducting the experiments. All procedures of this study were approved by the Institutional Animal Care and UseCommittee of Kyung Hee University (KHUASP(SE)-20-679).

### 4.5. Paclitaxel Administration

Paclitaxel (Sigma-Aldrich, St. Louis, MO, USA) was dissolved in 100% ethanol (Merck, Marmstadt, Germany) and chromoper EL solution (Sigma-Aldrich, St. Louis, MO, USA) at a concentration of 6 mg/mL to make stock. The stock solution was then diluted with phosphate-buffered saline (PBS) to a final concentration of 0.2 mg/mL when used. Paclitaxel was intraperitoneally administered to the mice every other day (days 0, 2, 4, and 6) with a total dose of 8 mg/kg (2 mg/kg/day). Control group mice were injected with vehicle (100% ethanol and chromoper EL) diluted with PBS [19,20,58].

### 4.6. Behavioral Assessment

To confirm the establishment of neuropathic pain, cold allodynia tests were performed before and after the paclitaxel administration. Mice were placed in an inverted transparent plastic cage (12 cm × 8 cm × 6 cm) on a metal mesh grid and acclimatized for 30 min. Before the test, the mice were acclimatized for 7 days so that they could get used to the behavioral experiment space and plastic cage. The experimenter proceeded to blindly conduct the drug administration [59,60]

In the cold allodynia test, 10 uL of acetone was applied on the center of the plantar surface of the hind paw, and then behavioral responses of licking or shaking the hind paw were observed for 30 s. Tests were repeated 3 times in each hind paw. The total counted licking and shaking behaviors in each hind paw were added up and divided by 6. The control group also experienced the same tests [21,59].

### 4.7. Experimental Schedule

The animals were acclimatized to the experimental space, plastic cage, and stimulation environments for 7 days. After this adaptation period, the baseline test was performed and the day was set as day 0. PTX was injected on day 0, 2, 4, and 6 [19]. Behavior tests were performed on day 0, 1, 4, 7, 14, and 24. The efficacy of magnolin was measured on a day between day 7 and day 14, a period when PTX-induced pain symptom was severe. On this day, animal behavior was measured before and 1, 2, and 4 h after the magnolin treatment. 

### 4.8. Western Blot

To compare the PTX-induced change, DRG samples of the PTX group and the vehicle group were collected on day 14 (14 days after the first injection of PTX or vehicle). To assess the change of protein expression following the magnolin treatment, DRG samples were collected 1 and 2 h after administration of magnolin in separate PTX group animals. After adding tissue lysis buffer (iNtRON Biotechnology, Seongnam-si, Gyeonggi-do, Korea) and beads to the collected sample, the tissue was homogenized and placed on ice for 1 h to lyse. The obtained protein was quantified by the Bradford assay method. An equal amount of protein was mixed with 10% β-mercaptoethanol and heated in a heat block at 100 °C for 10 min. Proteins (20 µg) were separated by 12% SDS-PAGE and transferred to a 0.2 um polyvinylidene fluoride (PVDF) membrane using an electrophoretic transfer system through transfer (Bio-Rad Laboratories, Hercules, CA, USA). After blocking the membrane with 5% BSA in PBS containing 0.1% Tween-20 (PBST) for 1 h at room temperature, samples were mixed with the primary antibody with 3% BSA in PBST (rabbit Anti-pERK (1:2000, Danvers, MA, Cell Signaling). The membrane was incubated overnight at 4 °C. After washing using PBST, the sample was incubated with rabbit IgG antibody (1:2000; Vector Laboratories, Burlingame, CA, USA) for 1 h. For normalization of antibody signals, the PVDF membranes were stripped and reprobed with rabbit Anti-pERK (1:2000, Cell Signaling). Western blot assay was performed at least three times. 

### 4.9. Data Analysis

Data were tested with the Shapiro-Wilk test and F test to confirm the normal distribution and the homogeneity of variances. Two-way repeated measures ANOVA followed by Sidak’s multiple comparisons test was used to compare the behavioral data in Figure 3 and Figure 4. In the comparison of protein expression, non-parametric method was adopted as the data of 1 h in Figure 5 could not pass the normality test (*p* = 0.0478). Kruskal-Wallis test followed by Dunn’s multiple comparisons test was used to compared the protein expression in Figure 5. Statistical analysis was performed using Prism v 7.0 (GraphPad Software, La Jolla, CA, USA). All data were presented as mean ± SEM. In all the cases, tests were considered significant at *p* < 0.05.

## 5. Conclusions

Three tetrahydrofurofuranoid lignans, magnolin, kobusin, and aschantin were isolated from the seeds of *M. denudata* as major constituents. Among the lignans, magnolin was selected and its efficacy for CIPN was investigated. Mice treated with PTX exhibited cold allodynia, a representing symptom of CIPN. Magnolin treatment successfully alleviated the PTX-induced cold allodynia, shown by a significant behavioral change in PTX mice treated with an intraperitoneal injection of 1 or 10 mg/kg magnolin. Following molecular analysis showed that the magnolin treatment could inhibit the phosphorylation of ERK1/2 in the DRG of PTX group mice. In conclusion, these data demonstrated the analgesic effect of magnolin on the PTX-induced cold allodynia symptom and the inhibitory effect of magnolin on the ERK1/2 phosphorylation in the DRG of PTX-treated mice. We propose magnolin as a substance that could be a novel candidate for the development of therapeutics for CIPN.

## 6. Patents

N.K., G.C., S.-R.S., D.S.J. and S.K.K. hold a patent related to the contents of this article (application #:10-2021-0150313 in Korea).

## Data Availability

The data for this study are available from the corresponding authors upon reasonable request.

## Supplementary Materials

The following supporting information can be downloaded at: https://www.mdpi.com/article/10.3390/plants12122283/s1, Figure S1. ^1^H-NMR spectrum for (+)-magnolin (500 MHz, CDCl_3_); Figure S2. ^13^C-NMR spectrum for (+)-magnolin (125 MHz, CDCl_3_); Figure S3. UHPLC-PDA-MS chromatogram for (+)-magnolin; Figure S4. ^1^H-NMR spectrum for kobusin (500 MHz, CDCl_3_); Figure S5. ^1^H-NMR spectrum for aschantin (500 MHz, CDCl_3_).
