# Supplementary material for: Magnolin Inhibits Paclitaxel-Induced Cold Allodynia and ERK1/2 Activation in Mice
Source: Plants (Basel). 2023 Jun 12;12(12):2283. doi: 10.3390/plants12122283 (PMC10303305; doi:10.3390/plants12122283)

Figure S1. ^{1}H-NMR spectrum for (+)-magnolin (500MHz, $CDCl_3$)

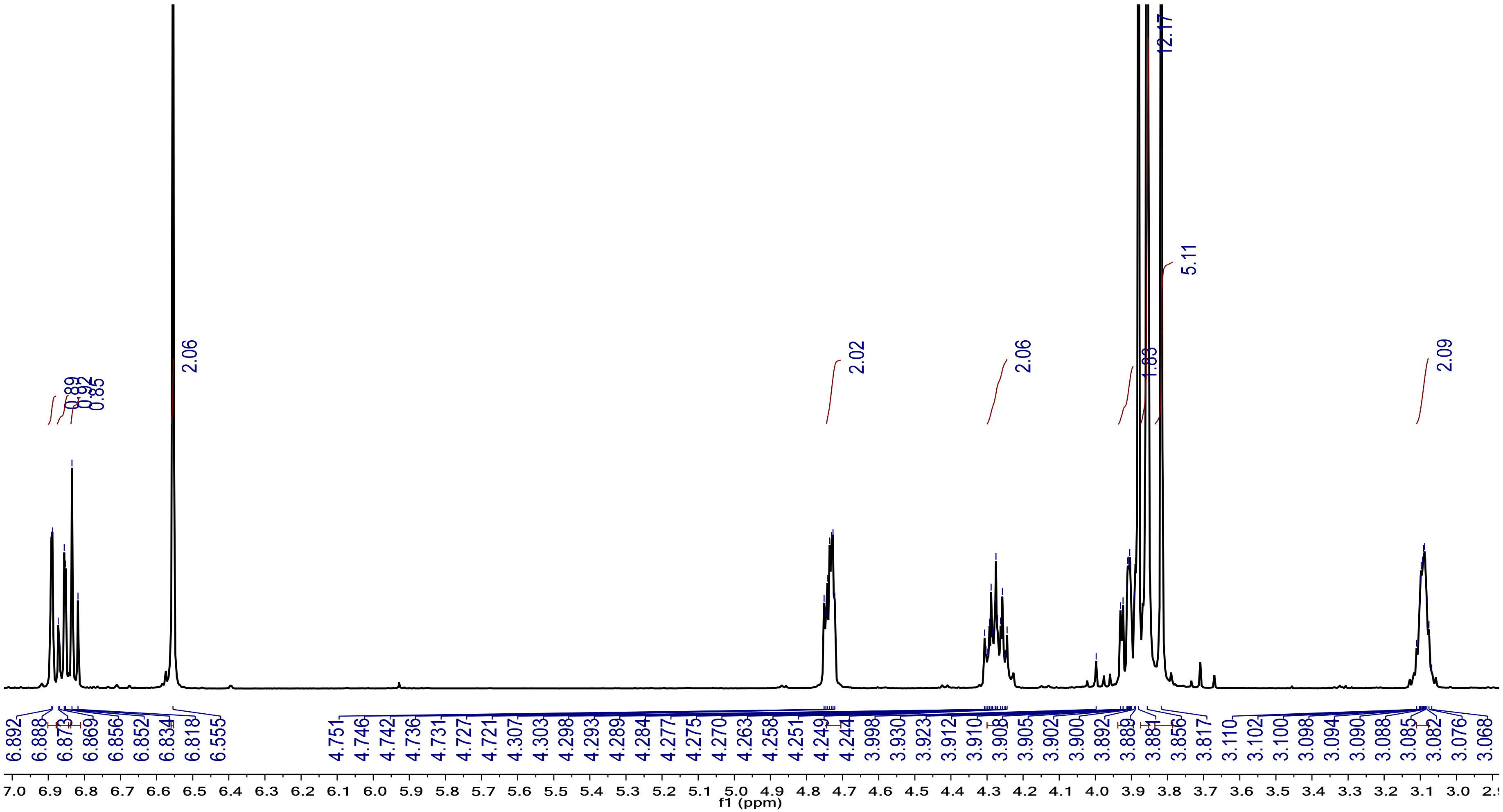

Figure S2. ^{13}C-NMR spectrum for (+)-magnolin (125MHz, $CDCl_3$)

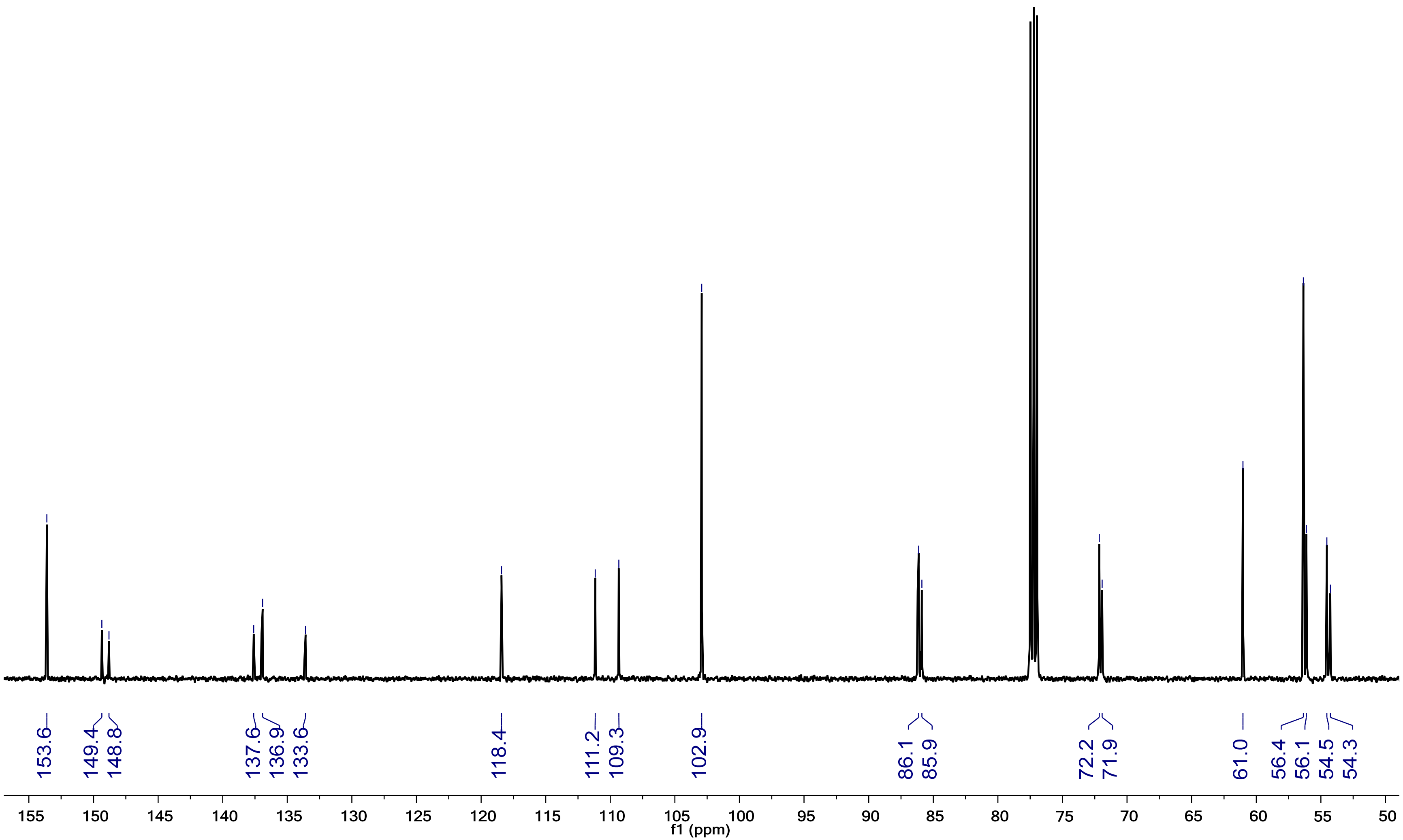

Figure S3. UHPLC-PDA-MS chromatogram for (+)-magnolin (RT 15.96 min); A: PDA chromatogram of (+)-magnolin, B: TIC chromatogram of (+)-magnolin, C: UV spectrum of (+)-magnolin, D: ESI Full MS scan of (+)-magnolin. A stock solution of 0.5 mg/mL was dissolved in methanol containing 20% DMSO. Data collected by Thermo Vanquish UHPLC system equipped with Thermo Hypersil GOLD column (1.9μm, 150 × 2.1 mm I.D.). The mobile phase was subjected to a series of linear gradients with a flow rate of 0.3 mL/min: 0–3min, 10% B; 3–35min, 100%B; 35–42min, 100%B; 42–43min 10%B.

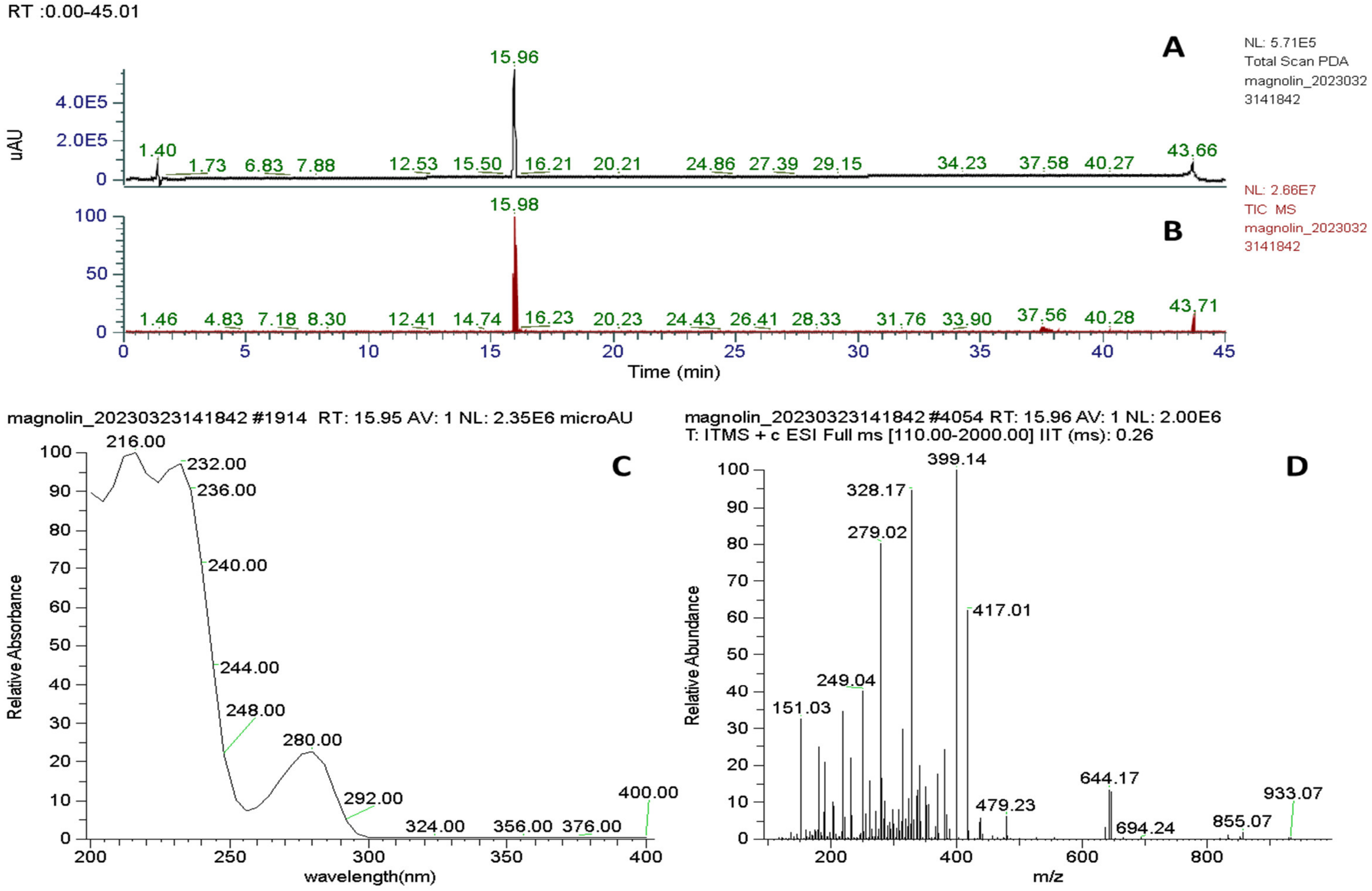

Figure S4. ^{1}H-NMR spectrum for kobusin (500MHz, $CDCl_3$)

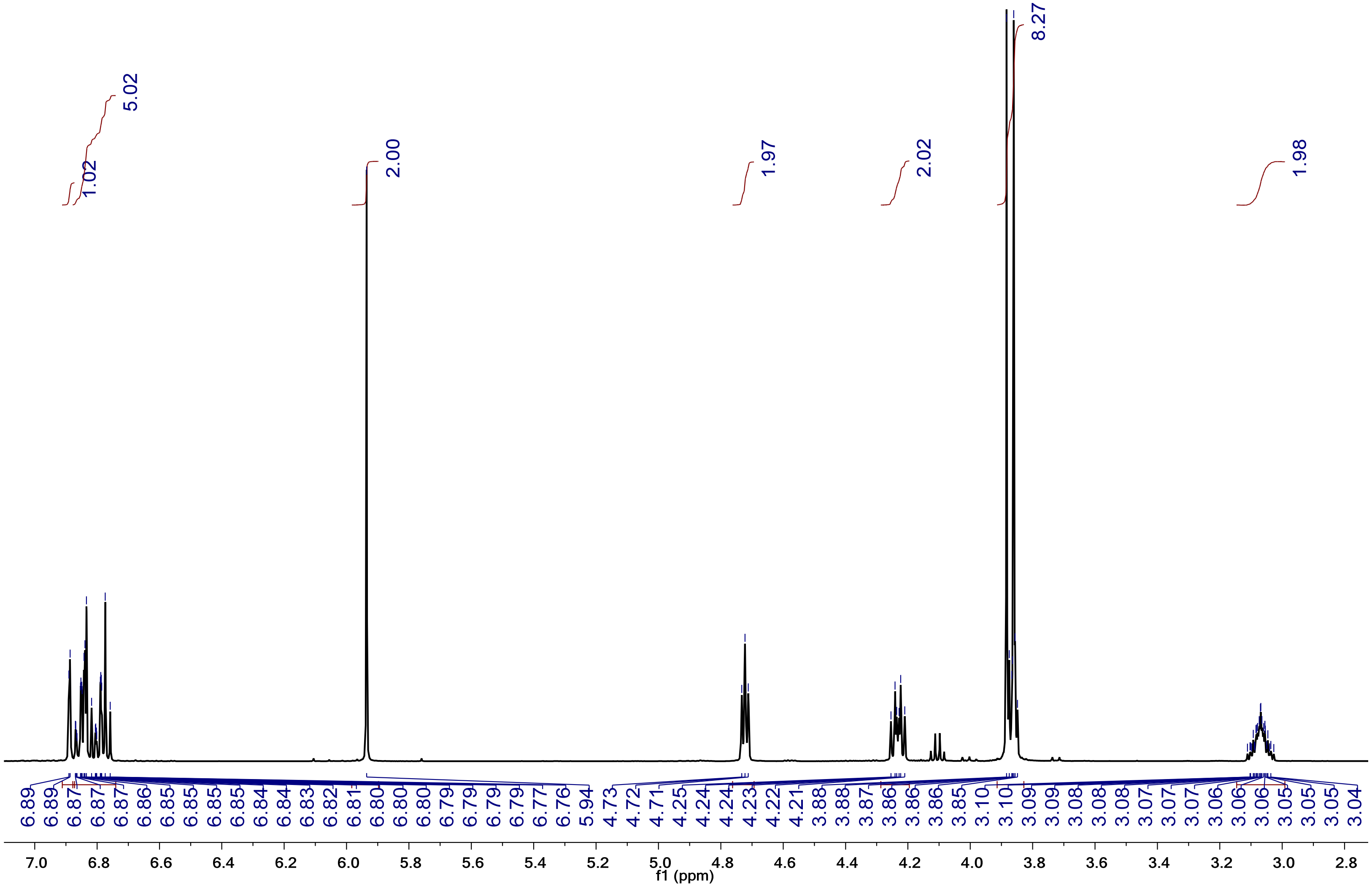

Figure S5. ^{1}H-NMR spectrum for aschantin (500MHz, $CDCl_3$)

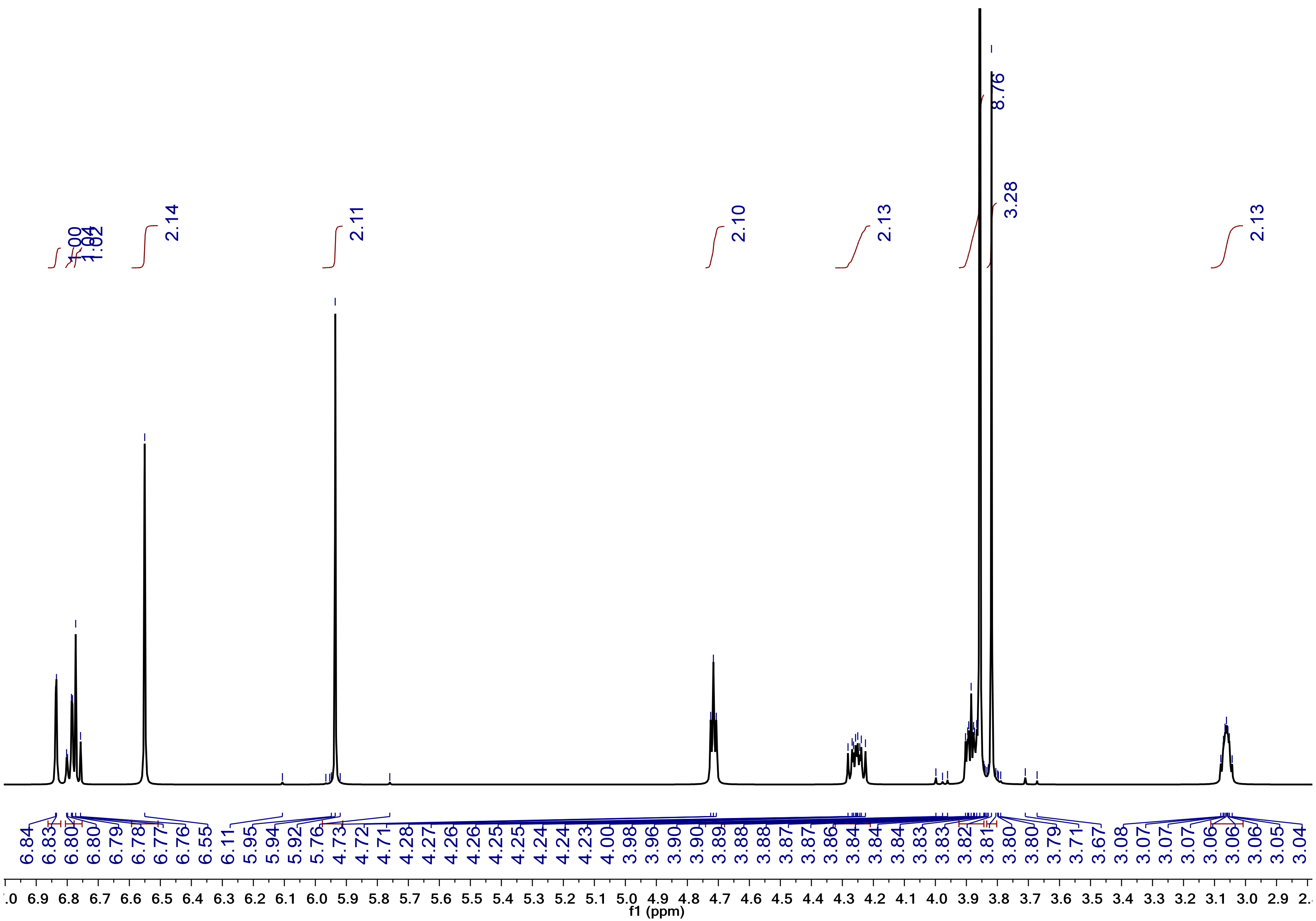

Supplement: Supplementary file 1 [file plants-12-02283-s001.zip › plants-2367379-supplementary.pdf]
